# Blunt Snout Bream (*Megalobrama amblycephala*) MyD88 and TRAF6: Characterisation, Comparative Homology Modelling and Expression

**DOI:** 10.3390/ijms16047077

**Published:** 2015-03-30

**Authors:** Ngoc Tuan Tran, Han Liu, Ivan Jakovlić, Wei-Min Wang

**Affiliations:** College of Fisheries, Key Lab of Agricultural Animal Genetics, Breeding and Reproduction of Ministry of Education/Key Lab of Freshwater Animal Breeding, Ministry of Agriculture, Huazhong Agricultural University, Wuhan 430070, China; E-Mails: tranntts@gmail.com (N.T.T.); lifegood1986@126.com (H.L.); ivanjakovlic@yahoo.com (I.J.)

**Keywords:** *Megalobrama amblycephala*, *Ma*MyD88, *Ma*TRAF6, physicochemical characterisation, homology modelling, gene expression

## Abstract

MyD88 and TRAF6 play an essential role in the innate immune response in most animals. This study reports the full-length *MaMyD88* and *MaTRAF6* genes identified from the blunt snout bream (*Megalobrama amblycephala*) transcriptome profile. *MaMyD88* is 2501 base pairs (bp) long, encoding a putative protein of 284 amino acids (aa), including the *N*-terminal DEATH domain of 78 aa and the *C*-terminal TIR domain of 138 aa. *MaTRAF6* is 2252 bp long, encoding a putative protein of 542 aa, including the *N*-terminal low-complexity region, RING domain (40 aa), a coiled-coil region (64 aa) and *C*-terminal MATH domain (147 aa). Coding regions of *MaMyD88* and *MaTRAF6* genomic sequences consisted of five and six exons, respectively. Physicochemical and functional characteristics of the proteins were analysed. Alpha helices were dominant in the secondary structure of the proteins. Homology models of the *Ma*MyD88 and *Ma*TRAF6 domains were constructed applying the comparative modelling method. RT-qPCR was used to analyse the expression of *MaMyD88* and *MaTRAF6* mRNA transcripts in response to *Aeromonas hydrophila* challenge. Both genes were highly upregulated in the liver, spleen and kidney during the first 24 h after the challenge. While *MyD88* and *TRAF6* have been reported in various aquatic species, this is the first report and characterisation of these genes in blunt snout bream. This research also provides evidence of the important roles of these two genes in the blunt snout bream innate immune system.

## 1. Introduction

The immune system protects an organism against diseases by identifying and eliminating the pathogen [1]. Innate immunity, which is an evolutionarily ancient system present in both invertebrates and vertebrates, is the first line of defence against infection and an internal stimulator for development of antigen-specific acquired immune response and homeostasis [1,2,3,4,5]. The innate immune response is triggered by germline-encoded pattern recognition receptors (PRRs) that are responsible for recognising conserved pathogen-associated molecular patterns of foreign stimuli, such as lipopolysaccharide or peptidoglycan of bacterial cell walls, β-1,3-glucan of fungal cell walls, double-stranded RNA of viruses and endogenous molecules released from damaged cells [5,6,7,8]. To date, four different classes of PRR families have been identified: toll-like receptors (TLRs), *C*-type lectin receptors (both transmembrane proteins), retinoic acid-inducible gene (RIG)-I-like receptors and NOD-like receptors (both cytoplasmic proteins) [8]. TLRs are responsible for detecting extracellular invading pathogens and intracellular endosomes and lysosomes [9], leading to the activation and recruitment of immune effectors and subsequent stimulation of antimicrobial responses [10]. TLRs are characterised by the *N*-terminal leucine-rich repeats and a transmembrane region followed by a cytoplasmic Toll/IL-1R homology (TIR) domain [8]. The TIR region is required for initiating downstream signalling by recruiting adaptors that contain a TIR domain: MyD88 (originally identified as myeloid differentiation primary response gene [11]), MyD88-adaptor-like, TIR domain-containing adaptor protein (inducing interferon-β (IFN-β)), TIR-domain-containing adaptor protein and TRIF-related adaptor molecule [12]. While TLR3 and TLR4 are unique in their ability to activate the induction of type I IFNs in MyD88-independent fashion [10,13], most of the TLRs seem to be absolutely dependent on MyD88 for all of their functions [5]. MyD88 stimulates the expression of pro-inflammatory genes, like tumour necrosis factor (TNF) and interleukin-1 (IL-1) through the activation of NF-κB [7,14]. MyD88 consists of a *C*-terminal TIR and an *N*-terminal DEATH domain [5,15]. The DEATH domain, a protein interaction module composed of a bundle of six alpha helices, interacts with the corresponding domain in IL-1R-associated kinases (IRAKs), promoting recruitment of downstream immune molecules, including tumour necrosis factor receptor-associated factor 6 (TRAF6) [16]. Activation of TRAF6 by IRAKs recruits the induction of downstream immune molecules that subsequently lead to activation of NF-κB, IRFs and induction of pro-inflammatory cytokines or antiviral genes [8]. TRAF6, the most evolutionarily ancient TRAF family member, is also the only TRAF participating in signal transduction of both the TNF receptor superfamily and the IL-1R/TLR superfamily, which play essential roles in innate immunity, adaptive immunity and bone homeostasis [17].

Both genes have been identified and their functions studied in a broad range of aquatic animals: such as Zhikong scallop (*Chlamys farreri*) [18], common carp (*Cyprinus carpio*) [19], miiuy croaker (*Miichthys miiuy*) [20], whiteleg shrimp (*Litopenaeus vannamei*) (MyD88) [21], penaeid shrimp (*Fenneropenaeus chinensis*) [22], zebrafish (*Danio rerio*) [23], orange-spotted grouper (*Epinephelus coioides*) [24], whiteleg shrimp (TRAF6) [25] and grass carp (*Ctenopharyngodon idella*) [26]. However, so far MyD88 and TRAF6 have not been reported in blunt snout bream (*Megalobrama amblycephala*). Based on the available data from other (related) species, we hypothesize that both genes are present in blunt snout bream and that their structure and functions are conserved. Herein, the transcripts of *MaMyD88* and *MaTRAF6* were identified from the transcriptomic profile of blunt snout bream and their expression levels after challenge with *Aeromonas hydrophila* were investigated. Additionally, the entire coding regions of *MaMyD88* and *MaTRAF6* genes were amplified and sequenced from the genomic DNA. Computational tools were used to analyse the physicochemical characteristics and predict the structure of the encoded proteins. This study provides an insight into the role of *MaMyD88* and *MaTRAF6* in antibacterial response mechanisms of the blunt snout bream immune system, which can facilitate the utilisation of molecular tools for selective breeding of disease-resistant blunt snout bream broodstock in order to reduce mortality and increase productivity during the cultivation.

## 2. Results

### 2.1. Sequence Analysis

**Figure 1:**
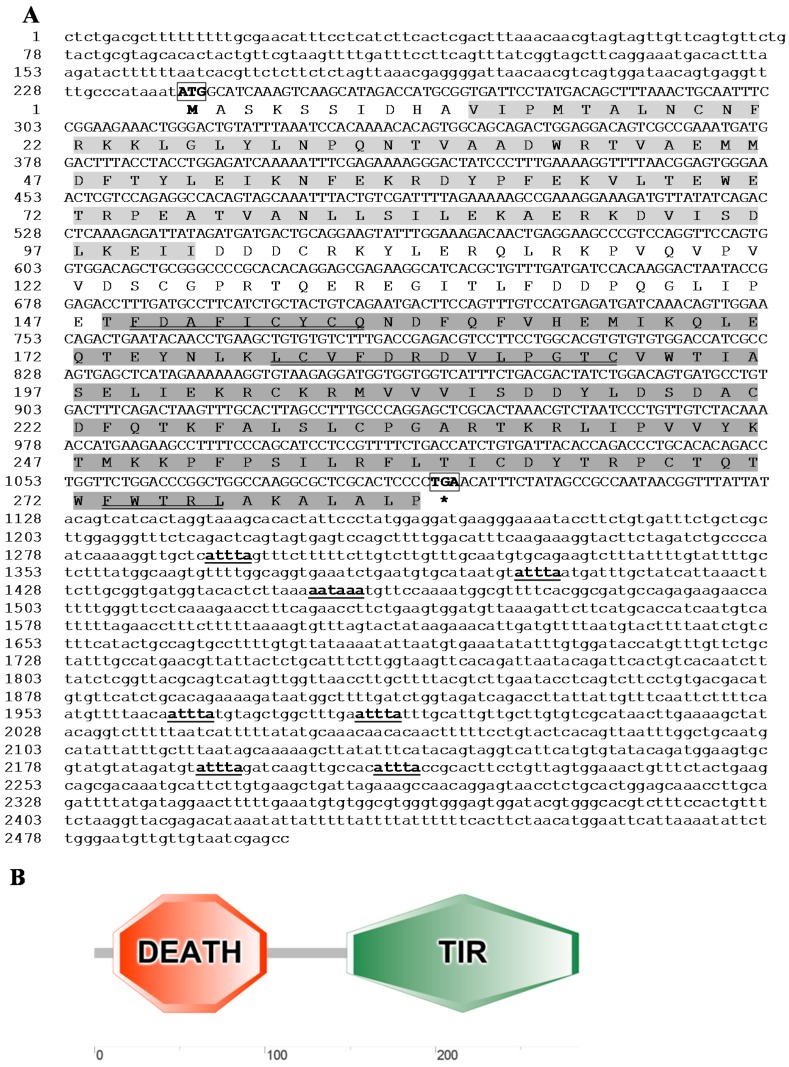
Sequences and domain topology of *Ma*MyD88 identified from the blunt snout bream transcriptome profile. (**A**) The nucleotide (upper row) and deduced amino acid (lower row) sequence are shown and numbered on the left. Start (ATG) and stop (TGA) codons are bolded and boxed. mRNA instability motifs (ATTTA) and a consensus polyadenylation signal sequence (AATAAA) are bolded and underlined. DEATH (Val11–Ile101) and TIR (Thr184–Pro284) domain are shaded in light- and dark-grey, respectively. The three highly conserved boxes within the TIR domain are double-underlined. Asterisk mark (*) indicates the stop codon; (**B**) The architecture of the domain topology of *Ma*MyD88 protein, rendered by Simple Molecular Architecture Research Tool (SMART), showing cytoplasmic Toll/IL-1R homology (TIR) domain and a protein interaction module named DEATH domain.

**Figure 2:**
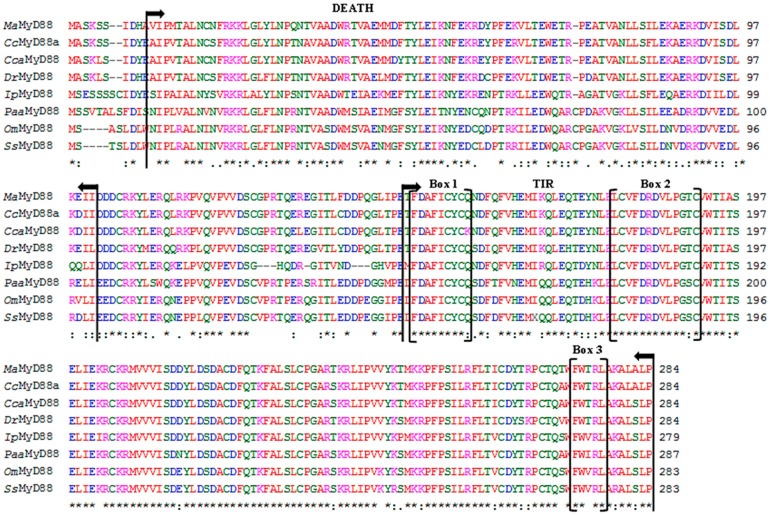
Multiple alignment of the deduced *Ma*MyD88 amino acid sequence with homologs from seven fish species: *Cyprinus carpio* (*Cc*MyD88a, Accession No. ADC45019.2), *Carassius carassius* (*Cca*MyD88, Acc. No. AGO57937.1), *Danio rerio* (*Dr*MyD88, Acc. No. NP_997979.2), *Ictalurus punctatus* (*Ip*MyD88, Acc. No. NP_001187207.1), *Plecoglossus altivelis altivelis* (*Paa*MyD88, Acc. No. BAI68385.1), *Oncorhynchus mykiss* (*Om*MyD88, Acc. No. CDG03206.1) and *Salmo salar* (*Ss*MyD88, Acc. No. NP_001130017.1). Asterisk marks (*) indicate identical amino acids. Sequences are numbered on the right, while the conserved substitutions are indicated by (:), semi-conserved substitutions by (.) and deletions by dashes. Arrows indicate DEATH and TIR domains and brackets indicate the three highly conserved boxes within the TIR domain.

The full *MaMyD88* transcript, deposited in NCBI GenBank database under the accession number KP192128, was 2501 base pairs (bp) long, containing a 239 bp 5' untranslated region (UTR), an open reading frame (ORF) of 855 bp encoding a putative protein of 284 amino acids (aa) and a 1407 bp 3' UTR. The 3' UTR contained a single typical polyadenylation signal (AATAAA) at the position (1454) and six mRNA instability motifs (ATTTA) (Figure 1A). Start (ATG) and stop (TGA) codons were determined from the 5' end of the sequence at the nucleotide positions 240 and 1092, respectively. Molecular weight of the putative protein was 33 kDa. The *N*-terminal DEATH domain consisting of 91 aa (Val11–Ile101) and the *C*-terminal TIR domain consisting of 137 aa (Thr184–Pro284) were found in the protein (Figure 1B). *N*-terminal signal peptide and *N*-linked glycosylation sites were not found. Three highly conserved regions (Box 1: ^149^FDAFICYCQ^157^, Box 2: ^179^LCVFDRDVLPGTC^191^ and Box 3: ^273^FWTRL^277^) were found within the TIR domain (Figure 1A). *Ma*MyD88 shares high amino acid sequence similarity (>60%) with homologs in other teleosts, particularly cyprinids: common carp (94%), crucian carp (*Carassius carassius*) (93%) and zebrafish (90%) (Figure 2).

**Figure 3:**
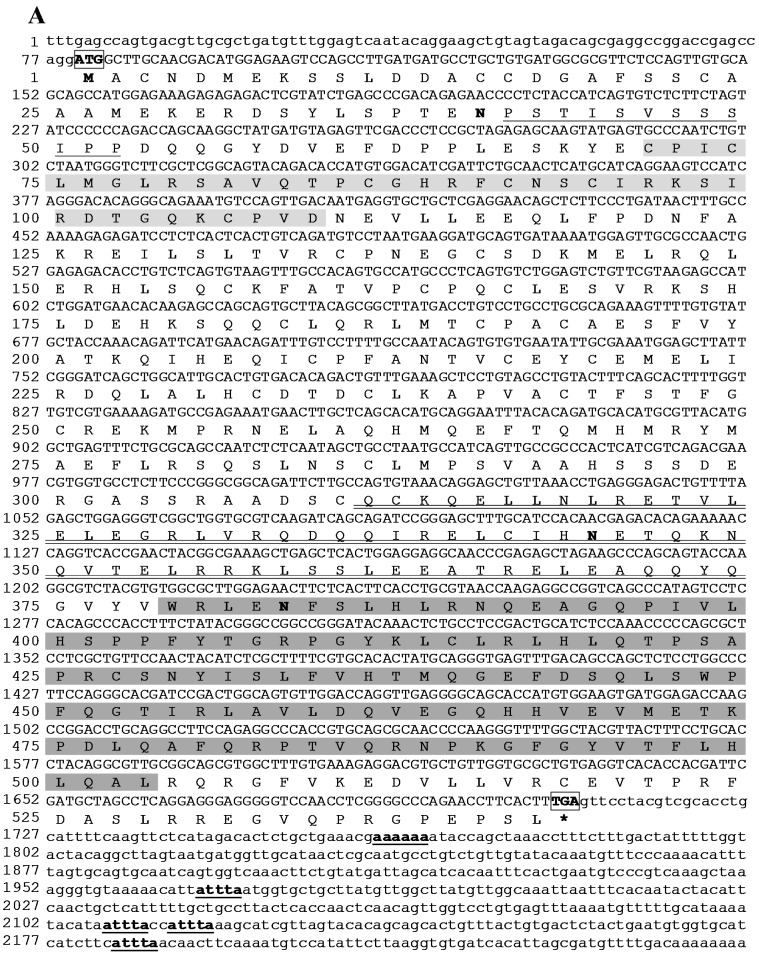

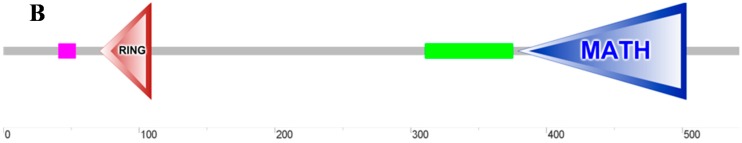
Sequence and domain topology of *Ma*TRAF6 identified from the blunt snout bream transcriptome profile. (**A**) The nucleotide (upper row) and deduced amino acid (lower row) sequence are numbered on the left. The potential *N*-linked glycosylation sites, start codon (ATG) and stop codon (TGA) are bolded and boxed. mRNA instability motifs (ATTTA) and the consensus polyadenylation signal sequence (AAAAAA) are bolded and underlined. The low complexity and coiled coil regions are single- and double-underlined, respectively. RING (Cys71–Asp109) and MATH (Trp379–Leu503) domains are shaded in light- and dark-grey, respectively. Asterisk mark (*) indicates the stop codon; (**B**) The architecture of the domain topology of *Ma*TRAF6 protein, rendered by Simple Molecular Architecture Research Tool (SMART), showing the low-complexity region (pink), zinc finger type protein structural domain (RING), coiled-coil (green) and the meprin and TRAF homology (MATH) domain.

The complete *MaTRAF6* transcript, deposited in the NCBI’s GenBank database under the accession number KP192129, was 2252 bp long, containing an 79 bp 5' UTR and a 547 bp 3' UTR. A polyadenylation signal sequence AAAAAA was found at the position 1762. Four mRNA instability motifs (ATTTA) were found in the 3' UTR. Three potential *N*-linked glycosylation sites (at positions 40, 344 and 383) were located in the coding region. The start (ATG) and stop (TGA) codons were determined from the 5' end of the cDNA at positions 80 and 1706, respectively. The ORF of 1629 bp is encoding a putative polypeptide chain of 542 aa with molecular weight of 61.8 kDa. Sequence analysis results revealed that *Ma*TRAF6 consists of a low complexity region of 12 aa (Pro41–Pro52), an *N*-terminal zinc finger type protein structural domain, named RING domain (Cys71–Asp109), a coiled-coil region (Gln311–Gln374) and a conserved *C*-terminal meprin and TRAF homology (MATH) domain (Trp379–Leu503) (Figure 3). No *N*-terminal signal peptide was found in the putative protein. *Ma*TRAF6 also shares high amino acid sequence similarity with TRAF6 proteins of other teleosts (>57%), particularly cyprinids: grass carp (99%), common carp (94%) and zebrafish (91%).

### 2.2. Genomic Organisation Analysis

The entire coding regions of the *MaMyD88* and *MaTRAF6* genes were amplified and sequenced from the genomic DNA. The analysis revealed that both are composed of a chain of exons, separated by introns. *MaMyD88* gene contains five exons (304, 123, 198, 91 and 155 bp) and 4 introns (590, 78, 1386, and 362 bp). The first exon encodes the DEATH domain, while exons 3, 4 and 5 encode the TIR domains. *MaTRAF6* gene sequence consists of six exons (299, 151, 159, 72, 78 and 867 bp), separated by five introns (501, 1438, 286, 128 and 395 bp). The RING domain and coiled-coil region are encoded by the first two exons, while the MATH domain is encoded by the sixth exon. While no differences were found for *MaTRAF6*, the coding regions of the genomic *MaMyD88* translate into a putative 289 aa protein, due to the insertion of four amino acids at the position 204 (^204^QVCL^207^).

**Figure 4:**
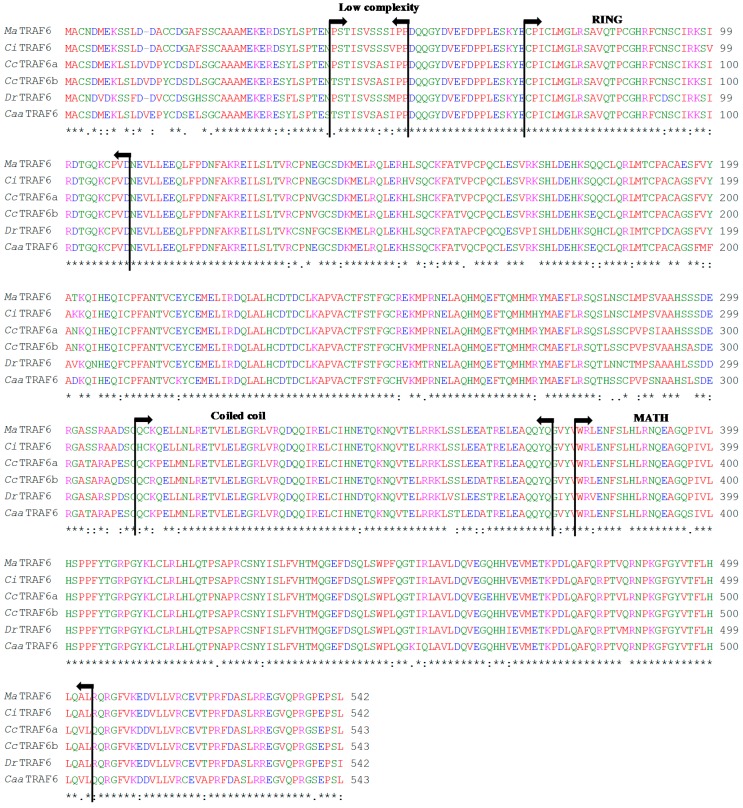
Multiple amino acid sequence alignment of *Ma*TRAF6 with TRAF6 homologs of five fish species: *Ctenopharyngodon idella* (*Ci*TRAF6, Acc. no. AGI51678.1), *Cyprinus carpio* (*Cc*TRAF6a, Acc. No. ADF56651.2 and ADM45856.1), *Danio rerio* (*Dr*TRAF6, Acc. no. NP_001038217.1) and *Carassius auratus auratus* (*Caa*TRAF6, Acc. No. AHG97567.1). Asterisk marks (*) indicate identical amino acids. Sequences are numbered on the right, conserved substitutions are indicated by (:), semi-conserved by (.) and deletions by dashes. Low complexity, zinc finger type protein structural domain (RING), coiled-coil and meprin and TRAF homology (MATH) domains are indicated by arrows.

### 2.3. Phylogenetic Analysis

**Figure 5:**
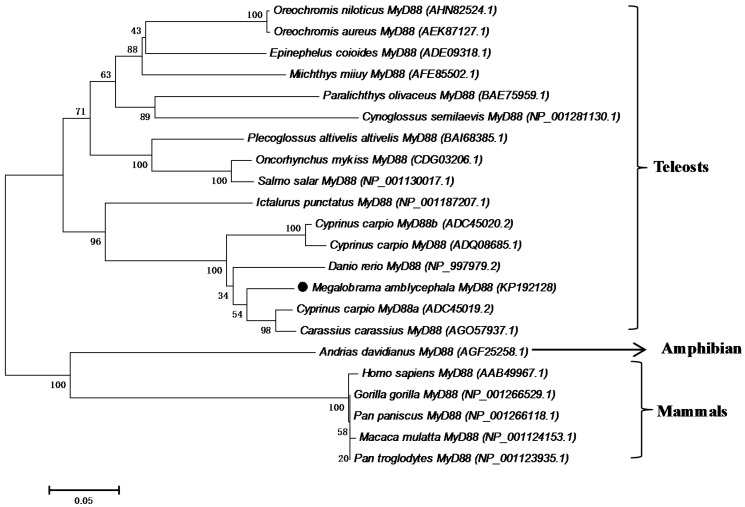
Neighbour-joining phylogenetic tree showing the relationships between the *Ma*MyD88 protein and homologs in selected animals (accession numbers in the parentheses). The numbers at the branches indicate bootstrap values (1000 replications). The bar (0.05) indicates the genetic distance. *Ma*MyD88 is marked by a black dot.

**Figure 6:**
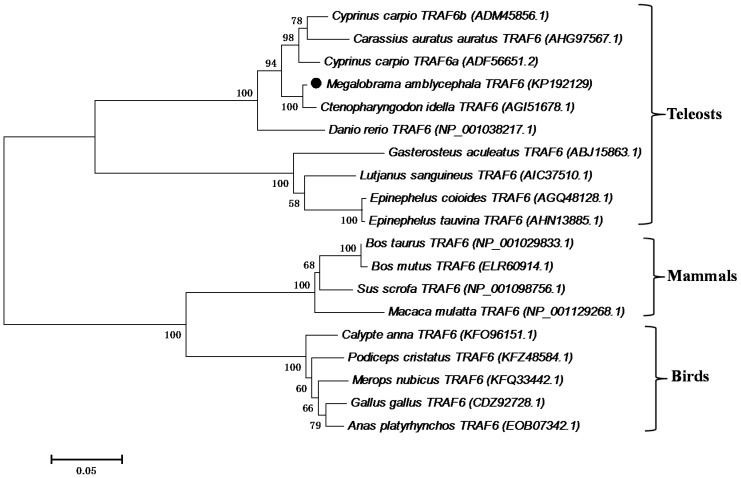
Neighbour-joining phylogenetic tree showing the relationships between the *Ma*TRAF6 and homologs in selected animals (accession numbers in the parentheses). The numbers at the branches indicate bootstrap values (1000 replications). The bar (0.05) indicates the genetic distance. *Ma*TRAF6 is marked by a black dot.

Both MyD88 and TRAF6 analyses revealed monophyletic teleost and cyprinid clusters, with *Ma*MyD88 and *Ma*TRAF6 very closely related to homologs from other cyprinids (Figure 5 and Figure 6). *Ma*MyD88 shares the highest similarity with common carp (ADC45019.2) and crucian carp (AGO57937.1) MyD88 sequences. *Ma*TRAF6, however, is the most similar to a grass carp (AGI51678.1) TRAF6 sequence.

### 2.4. Physicochemical and Functional Characterisation

Mature *Ma*MyD88 and *Ma*TRAF6 protein sequences without the signal peptide were used as the templates for physicochemical characterisation analyses (Table 1). The theoretical isoelectric point (pI) of both proteins was lower than 7, indicating they are acidic. As proteins carry a net positive charge below, and negative charge above their pI, this information can be used for the purification of proteins on a polyacrylamide gel by isoelectric focusing. The extinction coefficient (EC) of *Ma*MyD88 and *Ma*TRAF6 measured at 280 nm was 41,660 and 30,880 M^−1^·cm^−1^ (assuming all pairs of cysteine residues form cysteines) and 40,910 and 28,880 M^−1^ cm^−1^ (assuming all cysteine residues are reduced), respectively. The estimated half-life of both proteins is 30 h in mammalian reticulocytes *in vitro*, >20 h in yeast *in vivo* and >10 h in *Escherichia coli in vivo*. Based on the instability index (II) value, which is a measure to evaluate the stability of proteins in a test tube, *Ma*MyD88 protein (II = 33.5) is probably stable (II < 40), while *Ma*TRAF6 (II = 58.6) is probably not stable (II > 40) [27]. High AI (aliphatic index) value of *Ma*MyD88 (87.18) and *Ma*TRAF6 (72.14) indicate high thermostability of these proteins [28]. Low grand average hydropathicity (GRAVY) values of both proteins imply they are hydrophilic in natural conditions.

**Table 1:** Physicochemical characteristics of *Ma*MyD88 and *Ma*TRAF6 proteins: the number of amino acids (No. of aa), molecular weight in Da (Mol. Wt.), isoelectric point (pI), total number of negative (−R) and positive residues (+R), extinction coefficient (EC), instability index (II), aliphatic index (AI) and grand average hydropathicity (GRAVY).

Index	*Ma*MyD88	*Ma*TRAF6
No. of aa	284	542
Mol. Wt.	33,027.3	61,798.3
pI	5.89	5.91
−R	41	71
+R	39	60
EC *	41,660/40,910	30,880/28,880
II	33.5	58.6
AI	87.18	72.14
GRAVY	−0.241	−0.491

EC *—the first value is based on the assumption that all pairs of cysteine residues form cysteines and the second one that all cysteine residues are reduced.

*Ma*MyD88 protein is rich in leucine, aspartic acid, lysine and threonine, while *Ma*TRAF6 is rich in leucine, glutamic acid, and serine. Both proteins are classified as soluble. Twelve cysteine residues were found in *Ma*MyD88 and 32 in *Ma*TRAF6. The most probable pattern of cysteine residue pairing predicted in *Ma*MyD88 is Cys_105_–Cys_221_ and Cys_125_–Cys_268_, while no cysteine pairing was predicted in *Ma*TRAF6.

### 2.5. Protein Structure Prediction and Model Validation

In both proteins, alpha helix was predominant among the secondary structure elements, followed by random coil, extended strand and beta turn. The rest of the structure elements were not predicted (Table 2).

**Table 2:** Secondary structure elements (and % ratios) predicted by Self-Optimized Prediction Method with Alignment (SOPMA).

Element	*Ma*MyD88	*Ma*TRAF6
Alpha helix	44.37	40.96
3_10_ helix	0	0
Pi helix	0	0
Beta bridge	0	0
Extended strand	20.07	16.24
Beta turn	7.39	6.83
Bend region	0	0
Random coil	28.17	35.98
Ambiguous states	0	0
Other states	0	0

**Figure 7:**
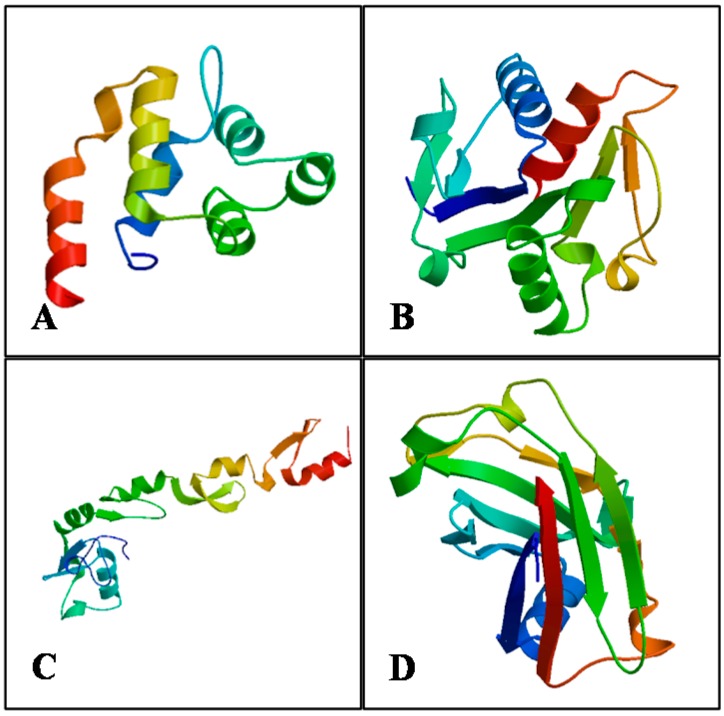
Three-dimensional structures of *Ma*MyD88 (**A**) DEATH and (**B**) TIR domains, and of *Ma*TRAF6 (**C**) RING and (**D**) MATH domains, predicted and rendered by SWISS-MODEL web server.

Based on the sequence and structural similarity, four different human protein structure templates available from the Protein Data Bank (PDB) were picked to build three-dimensional protein models (Figure 7): MyD88 (PDB ID: 3mop.1.C) [29] for the *Ma*MyD88 DEATH domain model (model coverage from Ile12 to Leu109; 44.9% sequence identity); MyD88 (PDB ID: 4eo7.1.A) [30] for the *Ma*MyD88 TIR domain (Pro146 to Pro284; 79.86%); *N*-terminal of TRAF6 (PDB ID: 3hcs.1.A) [31] for the *Ma*TRAF6 RING domain (Gln55 to Phe211; 67.09%) and TRAF6 (PDB ID: 1lb5.1.A) [18] for the *Ma*TRAF6 MATH domain (Gln371 to Pro522; 69.68%).

Ramachandran plot analysis results reveal that the models for TIR domain of *Ma*MyD88 and MATH domain of *Ma*TRAF6 have over 90% (92.2% and 90.8%, respectively) of residues in the most favoured region, suggesting a good quality of the homology models (Table 3). Both remaining models had almost all of the residues in most favoured and additional allowed regions combined (*Ma*MyD88 DEATH—96.7%, *Ma*TRAF6 RING—99.3%) indicating acceptable quality. The overall average G-factor of dihedral angles and main-chain covalent forces for the models ranged from −0.15 to 0.18, indicating a very good quality (>−0.5) of the proposed models [32]. LGscore values also indicate “very good” quality (>5.0), except for the *Ma*TRAF6 RING model (2.436), which is “correct” (>1.5). MaxSub validation measure indicates “very good” quality (>0.8) of the *Ma*MyD88 TIR domain, “correct” quality (>0.1) of *Ma*TRAF6 RING domain and “good” quality of the remaining two models (>0.5) [33]. The Z-Scores of the predicted models range from −7.63 to −5.24, which is within the range of the scores typically found for native proteins of the similar size, while plots of single residue energies revealed predominantly negative values, also indicating good quality of the proposed models [34] (data not shown). All of the validation results suggest that the proposed models of *Ma*MyD88 and *Ma*TRAF6 domains can be accepted as relatively accurate.

**Table 3:** Assessment of the predicted three-dimensional structures of the DEATH and TIR domains of *Ma*MyD88 protein, and RING and MATH domains of *Ma*TRAF6 protein, using different validation methods.

Validation Index	*Ma*MyD88		*Ma*TRAF6	
	DEATH	TIR	RING	MATH
Ramachandran plot				
Residues in most favoured regions	71.4%	92.2%	87.8%	90.8%
Residues in additional allowed regions	25.3%	7.8%	11.5%	7.7%
Residues in generously allowed regions	2.2%	0%	0.7%	0.8%
Residues in disallowed regions	1.1%	0%	0%	0.8%
Overall G-factor	−0.15	0.18	0.1	0.06
ProQ				
Lgscore	5.047	6.366	2.436	5.561
MaxSub	0.645	0.844	0.254	0.529
ProSA				
Z-Score	−5.24	−7.63	−5.32	−5.62

### 2.6. Expression of MaMyD88 and MaTRAF6 Transcripts after A. hydrophila Infection

**Figure 8:**
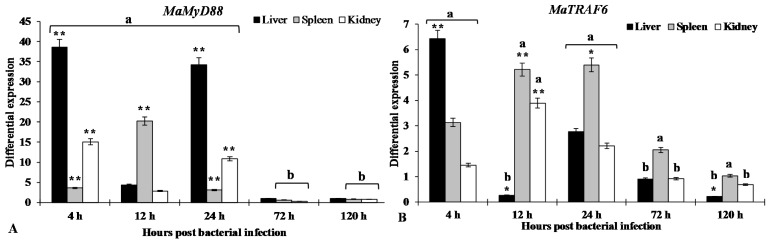
RT-qPCR results of gene expression profiles of *MaMyD88* (**A**) and *MaTRAF6* (**B**) in liver, spleen and kidney of blunt snout bream at 4, 12, 24, 72 and 120 h after challenge with *A. hydrophila*. Expression of genes in both control and experimental groups were normalised to the *18S* rRNA as a reference gene. The control group expression level is designated as 1, so values >1 indicate up-regulation (a), whereas values <1 indicate downregulation (b). Each histogram represents the mean ± SE of three replicates. Statistically significant differences from the control group are marked as * *p* < 0.05 and ** *p* < 0.01.

RT-qPCR was used to detect the changes in expression of *MaMyD88* and *MaTRAF6* in liver, spleen and kidney of blunt snout bream after *A. hydrophila* infection at different time points (4, 12, 24, 72, and 120 h post infection—hpi). In comparison to the control group (normalised to the internal control gene 18S rRNA expression levels), *MaMyD88* gene was significantly (*p* < 0.01) overexpressed in liver (38.6-fold) at 4 hpi, the expression was then reduced at 12 hpi (<5-fold), just to rise again significantly (>30-fold, *p* < 0.01) at 24 hpi. Expression in kidney followed a similar pattern: 15.9-fold (*p* < 0.01) at 4, <5-fold at 12 and >10-fold (*p* < 0.01) at 24 hpi. Expression in spleen was significantly (*p* < 0.01) upregulated at all three time-points, but the pattern was different: less than 5-fold at 4 hpi, 20.3-fold at 12 and then reverting to <5-fold level at 24 hpi. Expression at 72 and 120 hpi in all tissues was close to, or even below, the normal (control group) levels (Figure 8A).

The expression of *MaTRAF6* in liver followed a pattern somewhat similar to *MaMyD88*, albeit the over-expression levels were not nearly as high: significantly (*p* < 0.01) over-expressed (6.4-fold) at 4 hpi, significantly (*p* < 0.05) underexpressed at 12 hpi and then again over-expressed (2.8-fold) at 24 hpi. Expression then tailed off to the normal level at 72 hpi and to downregulated at 120 hpi (0.2-fold). Expression in kidney and spleen roughly followed a similar, bell curve pattern: peaking at 12 (3.9-fold, *p* < 0.01) and 24 (5.4-fold, *p* < 0.05) hpi, respectively, and then tailing off towards the normal level (Figure 8A).

## 3. Discussion

In this study, *MyD88* and *TRAF6* genes were identified and characterised in blunt snout bream for the first time. The genes were identified both from the transcriptome profile and genomic DNA. To test whether the functions of these genes are conserved in blunt snout bream, phylogenetic analysis was conducted, functional domains of amino acid sequences were predicted and characterised, secondary and tertiary structure of the proteins were predicted using computational tools and mRNA expression patterns in response to pathogen challenge were analysed. Genomic organisation analysis may also be helpful for further studies associated with identifying and developing gene polymorphisms related to disease susceptibility/resistance in blunt snout bream. Phylogenetic analyses suggest that both proteins are very similar to homologs from other cyprinids. There is even more divergence between different common carp MyD88 sequences, than between the common carp ADC45019 MyD88 and *Ma*MyD88 (Figure 5). While no differences were found between cDNA and genomic *MaTRAF6* sequences, genomic *MaMyD88* translates into a larger putative protein than cDNA due to insertion at the position 204 (^204^QVCL^207^). However, alignment with other sequences revealed the absence of this insertion (Figure 2), suggesting that the cDNA sequence is the correct protein template.

The presence of conserved functional domains in *Ma*MyD88 protein suggests that it probably has the same function (interaction with TLRs and IL-1R) as its homologs in other animals [26,28]. The DEATH domain plays important functions in DEATH signal transduction, regulation of apoptosis and inflammatory responses [35], whereas the TIR domain is important in activating the innate immune response by the TLR/IL-1R superfamily mediated pathway [36]. Three highly conserved box regions found within the TIR domain (Figure 1A) are in accordance with results reported in frogs, mammals and fish [28]: boxes 1 and 2 mediate the coupling to inflammation signalling pathways and box 3 is primarily related to the control of subcellular location of the receptor, possibly through interactions with cytoskeletal elements [37]. Furthermore, the presence of mRNA instability motif in the 3' UTR is characteristic of some fish inflammatory mediator-coding genes and is believed to be responsible for destabilising mRNA [27,38].

*Ma*TRAF6 primary and secondary structure is comparable to those reported in other fish species, such as zebrafish [23], common carp [19] and grass carp [26]. However, zinc fingers were seemingly absent from the *Ma*TRAF6 amino acid sequence. The RING and/or zinc finger regions of TRAFs are critical for signalling, probably due to their association with downstream kinase molecules such as MEKK1 [31]. When it was compared to the most similar, grass carp homolog (Figure 6), where two zinc fingers were reported: 152–204 and 205–262 aa [26], two aa mutations were found in the first zinc finger. At the position 195—glycine (G/GGG) in grass carp was changed to glutamic acid (E/GAA) in *Ma*TRAF6 and at the position 201—lysine (K/AAG) was changed to threonine (T/ACC). However, the second zinc finger appears identical in both species, suggesting that the reported differences could have actually been caused by different parameters used by the employed software, and not by different functional properties of the proteins. To further corroborate this, a common carp TRAF6 sequence, where no zinc fingers were reported [19], was also compared to the grass carp: only one mutation was found in the putative zinc finger one region (position 201) and none in the zinc finger two. Comparative analysis revealed that position 201 is the least conserved position in the cyprinid zinc finger one domain, suggesting its low relevance for the protein function. Similarly, only one zinc finger was reported in zebrafish, spanning aa 205 to 260 [23]. Only one mutation was found at the position 256, where threonine (T) was changed to proline (P/CCG) in *Ma*TRAF. Furthermore, *Ma*TRAF6 RING and MATH domains share very high similarity with the homologous domains in other teleosts (Figure 4). The coiled-coil region plays a key position in the auto-ubiquitination and activation of the NF-κB signalling pathway [39], while the MATH domain is responsible for binding TRAF6 to upstream molecules [17]. While seven exons and six introns were found in the genomic sequences of TRAF6 in orange-spotted grouper [24] and zebrafish [23], identical numbers of exons were reported in common carp [19] and blunt snout bream MyD88 (5) and TRAF6 (6) genes. All these results suggest highly conserved functions of the homologs.

As protein structure often reflects its functions [40], physicochemical analysis and prediction of protein structures can provide basic insights into the functions of proteins at the molecular level. In theory, secondary protein structure is directly related to the hydrogen bonding of α-helix and β-sheet, while random coils usually indicate the absence of a regular secondary structure [41]. The observed patterns of cysteine pairing in *Ma*MyD88 indicate that the protein contains disulphide bonds that are essential in the folding of the protein and responsible for stabilisation of protein structure [42]. Analysis of three-dimensional structure of proteins has previously revealed the structural differences between receptor recognition by TRAF6 and other TRAFs, providing insight into the mechanisms by which TRAF6 mediates several signalling cascades [17]. As there is no available data about tertiary structure of these proteins in blunt snout bream, or even in closely related fish species, this study provides the basis for the further research of functional properties of these genes in fish.

Regulation patterns of both *MaMyD88* and *MaTRAF6* in response to a challenge by *A. hydrophila* reported here suggest a response of the host’s immune system to bacterial infection, and thus are consistent with the hypothesis of their conserved function in the immune system. Similar patterns were also observed in other studied fish: miiuy croaker *MyD88* was significantly upregulated at different time points (from 12 to 72 hpi) in liver, kidney and spleen after a challenge with *Vibrio anguillarum* [20]; Yan *et al.* [28] reported that *MyD88* transcription was induced in orange-spotted grouper spleens eight hours after a challenge with Singapore grouper iridovirus; it was also highly expressed in all the examined tissues in large yellow croaker (*Pseudosciaena crocea*) in response to formalin-inactivated *Vibrio parahaemolyticus* challenge [43].

In agreement with results presented here, *TRAF6* expression in aquatic animals was also previously found to be highly responsive to pathogen challenges [16,24,25,26]. In grass carp, *TRAF6* was up-regulated in spleen and head kidney six hours after a challenge with *Ichthyophthirius multifiliis*, while the expression was significantly down-regulated in head kidney at 72 hpi [26]. Similar expression patterns were observed in some crustaceans as well: in whiteleg shrimp, TRAF6 was upregulated in gills and hepatopancreas at 3 hpi with white spot syndrome virus (WSSV), and then down-regulated and maintained at a low level until the last tested time-point (24 hpi) [25]; similarly, *TRAF6* was upregulated in tiger shrimp (*Penaeus monodon*) haemocytes and lymphoid organ at the late stages of WSSV and poly I:C infection [16]. These results strongly suggest that *MaMyD88* and *MaTRAF6* play key roles in the blunt snout bream innate immunity. A good understanding of the blunt snout bream immune system is necessary for the prevention of diseases relevant for the farming of this species, however, further studies of the specific functions of these two genes are needed in order to fully confirm these assumptions.

## 4. Experimental Section

### 4.1. Ethics Statement

All animals and experiments were conducted in accordance with the “Experimental Animal Regulation in Hubei Province”. The regulation was approved in the 16th plenary session the Standing Committee of People’s Congress of Hubei Province. The announcement of the Standing Committee of People’ Congress of Hubei Province is code No. 50 (issued date: 29 July 2005 and effective date: 1 October 2005).

### 4.2. Fish and Challenge Experiment

Healthy blunt snout bream specimens (average body weight: 27.3 ± 6.8 g) used in this study were collected from the farm in Tuanfeng, Huanggang, Hubei, China. Fish were maintained in a 1 m^3^ tank with aeration at about 28 °C for 2 weeks in the laboratory at the College of Fisheries, Huazhong Agricultural University. Fish were fed commercial pelleted feed twice a day. For the analysis of mRNA expression, fish were challenged with *A. hydrophila*. Fish were divided into two groups: 87 specimens in the control group and 151 specimens in the experimental group. Fish from both groups were intraperitoneally injected equal volumes (0.1 mL) of sterile physiological saline (control) and a bacterial suspension at 1.8 × 10^6^ cfu/mL (experimental). Six specimens from each group were sampled at 4, 12, 24, 72 and 120 hpi anesthetised in MS-222 (Sigma-Aldrich, Saint Louis, MO, USA) at 100 mg/L concentration and immediately killed. Liver, spleen and kidney were separately sampled from all specimens, immediately flash-frozen in liquid nitrogen and stored at −80 °C.

### 4.3. Total RNA Preparation and cDNA Synthesis

The total RNA was extracted from each sample with RNAisoPlus Reagent (Takara Bio Inc., Dalian, China), according to the manufacturer’s instructions. Quality and quantity of the extracted RNA were estimated using electrophoresis in 1% agarose gel and Nanodrop 2000 spectrophotometry (Thermo Scientific, Wilmington, DE, USA). Equal amounts of the total RNA of six specimens sampled at each time-point were pooled. cDNA library was synthesised using PrimeScript^®^ RT reagent Kit with gDNA Eraser (Takara Bio Inc., Dalian, China) following the manufacturer’s instructions, serially diluted 10-fold and used as the template for RT-qPCR.

### 4.4. Cloning of Full-Length cDNAs and Bioinformatics Analyses

The full-length cDNAs of *MaMyD88* and *MaTRAF6* were obtained from the blunt snout bream transcriptome profile, constructed using Solexa/Illumina technology. The unigenes were firstly obtained via the *de novo* assembling of short reads from the transcriptome of blood, liver, gill, intestine, spleen and kidney. Then, the transcripts were annotated and identified through BLAST homology search against the GenBank database [44]. The full-length cDNA sequences of *MaMyD88* and *MaTRAF6* were obtained using SMART™ RACE cDNA Amplification Kit (Takara Bio Inc., Dalian, China), following the manufacturer’s instructions. The specific primers are described in Table 4. Thermal conditions for RACE-PCR were as follows: 94 °C for 5 min; 30 cycles of 94 °C for 30 s, 65 °C for 30 s, 72 °C for 2 min; extension at 72 °C for 5 min. The 3'-RACE and 5'-RACE products were ligated into the pGEM-T Easy vector (Promega, Madison, WI, USA) for sequencing. The full-length cDNA sequences were assembled using the SeqMan software. The putative amino acid sequences were predicted using the NCBI’s Open Reading Frame Finder [45]; signal peptide was predicted by using the SignalP server [46]; the poly-A tail was predicted by using The GENSCAN Web Server at MIT [47] and the domain structures using SMART program [48]. Homologous MyD88 and TRAF6 amino acid sequences from other species were obtained from the NCBI database. Multiple-sequence alignment was performed using the ClustalW2 server [49]. Neighbour-joining phylogenetic trees were constructed using MEGA 5.2 [50], and the topological stability of the trees was evaluated by 1000 bootstrap replications.

### 4.5. Protein Physicochemical and Functional Characterisation

Expasy’s ProtParam prediction server [51] was employed to determine the physicochemical properties of *Ma*MyD88 (284 aa) and *Ma*TRAF6 (542 aa) polypeptide chains: molecular weight, amino acid composition, theoretical isoelectric point, total number of positive and negative residues, extinction coefficient, instability index, aliphatic index and grand average of hydropathicity. SOSUI [52] was used to identify the types of protein (soluble or membrane) and CYS_REC [53] to predict the presence of disulphide bonds and their bonding patterns.

### 4.6. Protein Structure Prediction

Secondary structure of *Ma*MyD88 and *Ma*TRAF6 was predicted using the SOPMA server [54], with default parameters (window width—17; similarity threshold—8; number of states—4), while three-dimensional homology models were constructed using the SWISS-MODEL server [55]. The modelled structures were selected on the basis of sequence identity with the Protein Data Bank (PDB) templates [56]. Stereochemical quality and accuracy of the predicted models were analysed using PROCHECK’s Ramachandran plot analysis [57,58], ERRAT [59], ProQ [34] and ProSA [35].

### 4.7. Quantitative Real-Time PCR (RT-qPCR) and Statistics

RT-qPCR was performed with SYBR^®^ Premix Ex Taq™ (Takara Bio Inc., Dalian, China) in a Rotor-Gene Q real-time PCR cycler (Qiagen, Dusseldorf, Germany). The total reaction volume of 10 mL contained 5 µL of SYBR^®^ Premix Ex Taq II (2×), 0.4 µL of each primer (10 µM), 0.8 µL of cDNA template and 3.4 µL of dH_2_O. The primers (Table 4) were designed using Primer Premier 5 software (Premier Biosoft, Palo Alto, CA, USA) and synthesised by Sangon Biotech Co., Ltd. (Shanghai, China). 18S rRNA was used as the reference gene [60]. Thermal conditions were as follows: denaturation at 95 °C for 30 s, followed by 40 cycles of 95 °C for 5 s, annealing temperature at 55 °C—*MaMyD88*/53 °C—*MaTRAF6*/60 °C—*18S* rRNA for 20 s and elongation at 72 °C for 15 s. All reactions were performed in triplicate. The data were analysed using the Rotor-Gene Q series software 1.7 (build 94) (Qiagen). The *C*t values were obtained using a threshold value of 0.05 for all three genes. Relative gene expression was quantified by the comparative *C*t method, expressed as 2^−ΔΔ*C*t^ [61]. The relative expression of the target genes was normalised to an endogenous reference (*18S* rRNA), where Δ*C*t was calculated as *C*t_Test_ − *C*t_18S rRNA_, and ΔΔ*C*t was calculated as Δ*C*t_Test_ − Δ*C*t_Control_. The RT-qPCR data were analysed statistically with Microsoft Excel and with one-way analysis of variance (ANOVA) in the SPSS 16.0 software. Differences were considered statistically significant at *p* < 0.05 and *p* < 0.01.

**Table 4:** Primers used in this study.

Gene	Primer Sequence (5'–3')
*Primers used for RACE-PCR*	
*MaMyD88* 3'-RACE	GAGTCTGAGAAACCCTCCAAGCGA
*MaMyD88* 5'-RACE	AGGTGTAAGAGGATGGTGGTGGTC
*MaTRAF6* 3'-RACE	CAGTGACGTTGCGCTGATGTTTG
*MaTRAF6* 5'-RACE	GTCCCTGATGGACTTCCTGATGC
*Primers used for RT-qPCR*	
*MaMyD88*-F	GACAACAGGGATTAGACG
*MaMyD88*-R	TGGAACAGACTGAATACAAC
*MaTRAF6*-F	CGAGCGAAGACCCATTAGAC
*MaTRAF6*-R	ATCTGAGCCCGACAGAGAAC
*18S rRNA*-F	CGGAGGTTCGAAGACGATCA
*18S rRNA*-R	GGGTCGGCATCGTTTACG

## References

[1] Magnadottir B. (2010). Immunological control of fish diseases. Mar. Biotechnol..

[2] Tort L., Balasch J.C., Mackenzie S. (2003). Fish immune system. A crossroads between innate and adaptive responses. Inmunología.

[3] Whyte S.K. (2007). The innate immune response of finfish—A review of current knowledge. Fish Shellfish Immun..

[4] Basu M., Swai B., Maiti N.K., Routray P., Samanta M. (2012). Inductive expression of toll-like receptor 5 (TLR5) and associated downstream signaling molecules following ligand exposure and bacterial infection in the Indian major carp, mrigal (*Cirrhinus mrigala*). Fish Shellfish Immun..

[5] Takeda K., Akira S. (2005). Toll-like receptors in innate immunity. Int. Immunol..

[6] Medzhitov R., Janeway J.C. (2000). Innate immune recognition: Mechanisms and pathways. Immunol. Rev..

[7] Janeway C.A., Medzhitov R. (2002). Innate immune recognition. Annu. Rev. Immunol..

[8] Takeuchi O., Akira S. (2010). Pattern recognition receptors and inflammation. Cell.

[9] Akira S., Uematsu S., Takeuchi O. (2006). Pathogen recognition and innate immunity. Cell.

[10] Krishnan J., Selvarajoo K., Tsuchiya M., Lee G., Choi S. (2007). Toll-like receptor signal transduction. Exp. Mol. Med..

[11] Lord K.A., Hoffman-Liebermann B., Liebermann D.A. (1990). Nucleotide sequence and expression of a cDNA encoding MyD88, a novel myeloid differentiation primary response gene induced by IL6. Oncogene.

[12] Kawai T., Akira S. (2007). Signaling to NF-κB by toll-like receptors. Trends Mol. Med..

[13] Kawai T., Takeuchi O., Fujita T., Inoue J., Muhlradt P.F., Sato S., Hoshino K., Akira S. (2001). Lipopolysaccharide stimulates the MyD88-independent pathway and results in activation of IFN-regulatory factor 3 and the expression of a subset of lipopolysaccharide-inducible genes. J. Immunol..

[14] Medzhitov R., Preston-Hurlburt P., Kopp E., Stadlen A., Chen C., Ghosh S., Janeway C.A. (1998). MyD88 is an adaptor protein in the hToll/IL-1 receptor family signaling pathways. Mol. Cell.

[15] Wesche H., Henzel W.J., Shillinglaw W., Li S., Cao Z. (1997). MyD88: An adapter that recruits IRAK to the IL-1 receptor complex. Immunity.

[16] Deepika A., Sreedharan K., Paria A., Makesh M., Rajendran K.V. (2014). Toll-pathway in tiger shrimp (*Penaeus monodon*) responds to white spot syndrome virus infection: Evidence through molecular characterisation and expression profiles of MyD88, TRAF6 and TLR genes. Fish Shellfish Immun..

[17] Ye H., Arron J.R., Lamothe B., Cirilli M., Kobayashi T., Shevde N.K., Segal D., Dzivenu O., Vologodskaia M., Yim M. (2002). Distinct molecular mechanism for initiating TRAF6 signalling. Nature.

[18] Qiu L., Song L., Yu Y., Xu W., Ni D., Zhang Q. (2007). Identification and characterization of a myeloid differentiation factor 88 (MyD88) cDNA from Zhikong scallop *Chlamys farreri*. Fish Shellfish Immun..

[19] Kongchum P., Hallerman E.M., Hulata G., David L., Palti Y. (2011). Molecular cloning, characterization and expression analysis of TLR9, MyD88 and TRAF6 genes in common carp (*Cyprinus carpio*). Fish Shellfish Immun..

[20] Tang D., Gao Y., Wang R., Sun Y., Xu T. (2012). Characterization, genomic organization, and expression profiles of MyD88, a key adaptor molecule in the TLR signaling pathways in miiuy croaker (*Miichthys miiuy*). Fish Physiol. Biochem..

[21] Zhang S., Li C.Z., Yan H., Qiu W., Chen Y.G., Wang P.H., Weng S.P., He J.G. (2012). Identification and function of Myeloid Differentiation Factor 88 (MyD88) in *Litopenaeus vannamei*. PLoS ONE.

[22] Wen R., Li F., Sun Z., Li S., Xiang J. (2013). Shrimp MyD88 responsive to bacteria and white spot syndrome virus. Fish Shellfish Immun..

[23] Phelan P.E., Mellon M.T., Kim C.H. (2005). Functional characterization of full-length TLR3, IRAK-4, and TRAF6 in zebrafish (*Danio rerio*). Mol. Immunol..

[24] Li Y.W., Li X., Xiao X.X., Zhao F., Luo X.C., Dan X.M., Li A.X. (2014). Molecular characterization and functional analysis of TRAF6 in orange-spotted grouper (*Epinephelus coioides*). Dev. Comp. Immunol..

[25] Wang P.H., Wan D.H., Gu Z.H., Deng X.X., Weng S.P., Yu X.Q., He J.G. (2011). *Litopenaeus vannamei* tumor necrosis factor receptor-associated factor 6 (TRAF6) responds to *Vibrio alginolyticus* and white spot syndrome virus (WSSV) infection and activates antimicrobial peptide genes. Dev. Comp. Immunol..

[26] Zhao F., Li Y.W., Pan H.J., Wu S.Q., Shi C.B., Luo X.C., Li A.X. (2013). Grass carp (*Ctenopharyngodon idella*) TRAF6 and TAK1: Molecular cloning and expression analysis after *Ichthyophthirius multifiliis* infection. Fish Shellfish Immun..

[27] Guruprasad K., Reddy B.V.P., Pandit M.W. (1990). Correlation between stability of a protein and its dipeptide composition: A novel approach for predicting *in vivo* stability of a protein from its primary sequence. Prot. Eng..

[28] Yan Y., Cui H.C., Wei J.G., Huang Y.H., Huang X.H., Qin Q.W. (2012). Functional genomic studies on an immune- and antiviral-related gene of MyD88 in orange-spotted grouper, *Epinephelus coioides.*. Chin. Sci. Bull..

[29] Lin S.C., Lo Y.C., Wu H. (2010). Helical assembly in the MyD88-IRAK4-IRAK2 complex in TLR/IL-1R signalling. Nature.

[30] Snyder G.A., Cirl C., Jiang J., Chen K., Waldhuber A., Smith P., Römmler F., Snyder N., Fresquez T., Dürr S. (2013). Molecular mechanisms for the subversion of MyD88 signaling by TcpC from virulent uropathogenic *Escherichia coli*. Proc. Natl. Acad. Sci. USA.

[31] Yin Q., Lin S.C., Lamothe B., Lu M., Lo Y., Hura G., Zheng L., Rich R.L., Campos A.D., Myszka D.G. (2009). E2 interaction and dimerization in the crystal structure of TRAF6. Nat. Struct. Mol. Biol..

[32] Xu Y., Tao X., Shen B., Horng T., Medzhitov R., Manley J.L., Tong L. (2000). Structural basis for signal transduction by the toll/interleukin-1 receptor domains. Nature.

[33] Cristobal S., Zemla A., Fischer D., Rychlewski L., Elofsson A. (2001). A study of quality measures for protein threading models. BMC Bioinform..

[34] Wiederstein M., Sippl M.J. (2007). ProSA-web: Interactive web service for the recognition of errors in three-dimensional structures of proteins. Nucleic Acids Res..

[35] Weber C.H., Vincenz C. (2001). The death domain superfamily: A tale of two interfaces?. Trends Biochem. Sci..

[36] Janssens S., Beyaert R. (2003). Functional diversity and regulation of different interleukin-1 receptor-associated kinase (IRAK) family members. Mol. Cell.

[37] Slack J.L., Schooley K., Bonnert T.P., Mitcham J.L., Qwarnstrom E.E., Sims J.E., Dower S.K. (2000). Identification of two major sites in the type I Interleukin-1 receptor cytoplasmic region responsible for coupling to pro-inflammatory signaling pathways. J. Biol. Chem..

[38] Sachs A.B. (1993). Messenger RNA degradation in eukaryotes. Cell.

[39] Yang K., Zhu J., Sun S., Tang Y., Zhang B., Diao L., Wang C. (2004). The coiled-coil domain of TRAF6 is essential for its auto-ubiquitination. Biochem. Biophys. Res. Commun..

[40] Banerjee A.K., Arora N., Murty U.S.N. (2012). Analyzing a potential drug target *N*-Myristoyltransferase of *Plasmodium falciparum* through *in silico* approaches. J. Glob. Infect. Dis..

[41] Cai S., Singh B.R. (1999). Identification of β-turn and random coil amide III infrared bands for secondary structure estimation of proteins. Biophys. Chem..

[42] Hogg P.J. (2003). Disulfide bonds as switches for protein function. Trends Biochem. Sci..

[43] Yao C.L., Kong P., Wang Z.Y., Ji P.F., Liu X.D., Cai M.Y., Han X.Z. (2009). Molecular cloning and expression of MyD88 in large yellow croaker, *Pseudosciaena crocea*. Fish Shellfish Immun..

[44] Blast assembled genome. http://blast.ncbi.nlm.nih.gov/Blast.cgi.

[45] ORF Finder (Open Reading Frame Finder). http://www.ncbi.nlm.nih.gov/gorf/orfig.cgi.

[46] SignalP 4.1 Server. http://www.cbs.dtu.dk/services/SignalP/.

[47] The GENESCAN Web Server at MIT. http://genes.mit.edu/GENSCAN.html.

[48] SMART (Simple Modular Architecture Research Tool). http://smart.embl-heidelberg.de/.

[49] ClustalW2. http://www.ebi.ac.uk/Tools/msa/clustalw2/.

[50] Tamura K., Peterson D., Peterson N., Stecher G., Nei M., Kumar S. (2011). MEGA5: Molecular evolutionary genetics analysis using maximum likelihood, evolutionary distance, and maximum parsimony methods. Mol. Biol. Evol..

[51] Expasy’s ProtParam Tool. http://web.expasy.org/protparam/.

[52] Hirokawa T., Boon-Chieng S., Mitaku S. (1998). SOSUI: Classification and secondary structure prediction system for membrane proteins. Bioinformatics.

[53] Softberry. http://linux1.softberry.com/.

[54] Geourjon C., Deleage G. (1995). SOPMA: Significant improvements in protein secondary structure prediction by consensus prediction from multiple alignments. Comput. Appl. Biosci..

[55] Schwede T., Kopp J., Guex N., Peitsch M.C. (2003). SWISS-MODEL: An automated protein homology-modeling server. Nucleic Acids Res..

[56] Fiser A. (2010). Template-based protein structure modeling. Methods Mol. Biol..

[57] Ramachandran G.N., Ramakrishnan C., Sasisekhran V. (1963). Stereochemistry of polypeptide chain configuarations. J. Mol. Biol..

[58] Laskowski R.A., Rullmannn J.A., MacArthur M.W., Kaptein R., Thornton J.M. (1996). AQUA and PROCHECK-NMR: Programs for checking the quality of protein structures solved by NMR. J. Biomol. NMR.

[59] Colovos V.C., Yeates T.O. (1993). Verification of protein structures: Patterns of non-bonded atomic interactions. Protein Sci..

[60] Luo W., Zhang J., Wen J.F., Liu H., Wang W.M., Gao Z.X. (2014). Molecular cloning and expression analysis of major histocompatibility complex class I, IIA and IIB genes of blunt snout bream (*Megalobrama amblycephala*). Dev. Comp. Immunol..

[61] Livak K.J., Schmittgen T.D. (2001). Analysis of relative gene expression data using real-time quantitative PCR and the 2^−ΔΔ*C*t^ method. Methods.

